# Participation in physical play and leisure: developing a theory- and evidence-based intervention for children with motor impairments

**DOI:** 10.1186/1471-2431-11-100

**Published:** 2011-11-07

**Authors:** Niina Kolehmainen, Jillian J Francis, Craig R Ramsay, Christine Owen, Lorna McKee, Marjolijn Ketelaar, Peter Rosenbaum

**Affiliations:** 1 Health Services Research Unit, University of Aberdeen 3rd floor, Health Sciences Building, Foresterhill, Aberdeen, AB25 2ZD, UK; 2 Health Services Research Unit and Aberdeen Health Psychology Group, University of Aberdeen 3rd floor, Health Sciences Building, Foresterhill, Aberdeen, AB25 2ZD, UK; 3 Children's Occupational Therapy Service, St Johns Hospital, West Lothian, UK; 4 Rudolf Magnus Institute of Neuroscience and Center of Excellence for Rehabilitation Medicine, University Medical Center Utrecht and Rehabilitation Center De Hoogstraat, Rembrandtkade 10, 3583 TM Utrecht, The Netherlands; 5 Faculty of Health Sciences, Institute for Applied Health Sciences, Rm 408, 1400 Main Street West, Hamilton, Ontario, L8S 1C7, Canada

## Abstract

**Background:**

Children with motor impairments (e.g. difficulties with motor control, muscle tone or balance) experience significant difficulties in participating in physical play and leisure. Current interventions are often poorly defined, lack explicit hypotheses about why or how they might work, and have insufficient evidence about effectiveness. This project will identify (i) the 'key ingredients' of an effective intervention to increase participation in physical play and leisure in children with motor impairments; and (ii) how these ingredients can be combined in a feasible and acceptable intervention.

**Methods/Design:**

The project draws on the WHO International Classification of Functioning, Disability and Health and the UK Medical Research Council guidance for developing 'complex interventions'. There will be five steps: 1) identifying biomedical, personal and environmental factors proposed to predict children's participation in physical play and leisure; 2) developing an explicit model of the key predictors; 3) selecting intervention strategies to target the predictors, and specifying the pathways to change; 4) operationalising the strategies in a feasible and acceptable intervention; and 5) modelling the intervention processes and outcomes within single cases.

**Discussion:**

The primary output from this project will be a detailed protocol for an intervention. The intervention, if subsequently found to be effective, will support children with motor difficulties to attain life-long well-being and participation in society. The project will also be an exemplar of methodology for a systematic development of non-drug interventions for children.

## Background

Participation, including engagement in physical play and leisure, is fundamentally important to children's healthy development. It enables them to develop the social and physical competencies required to flourish, and provides social-emotional well-being, sense of meaning, and purpose in life [1]. Children with motor impairments (e.g. difficulties with motor control, muscle tone or balance) experience significantly reduced participation in leisure in general [2] and in physical play and leisure specifically [3]. These children constitute 6-9% of the population under 16 years of age [4] and are at life-long high risk of health and social difficulties [5,6]. Interventions in early life that effectively promote participation in physical play and leisure could provide considerable lifetime gains for children and their families, and, through preventing ill health, could bring cost savings to the health service and society more broadly.

Current interventions for children with motor impairments are so-called 'therapeutic activities' (e.g. games and exercises) recommended by occupational therapists and physiotherapists. The interventions are provided by therapists, parents and/or teachers in a variety of community settings (e.g. clinics, schools, homes). The interventions aim to increase what the children *can do*, including the range and quality of their pursuits and play. Most current interventions are believed to work through neurological, physiological and cognitive pathways that are thought to reduce impairment and/or develop skills. There is little high-quality research concerning most of these interventions and little empirical evidence to support their effectiveness [7-9].

Research in chronic pain [10] and stroke [11] has shown that behavioural interventions can be used to increase what an individual *can do *as well as what they *actually do *in real life. The approach involves supporting people to change behaviour using techniques that change beliefs (i.e. perceptions, expectations, etc) or behavioural regulation related to that behaviour. An example is using self-monitoring (a behaviour change technique) to increase a child's confidence (a belief about capability) in playing ball games (a behaviour). Some of these techniques are implicit in current treatment approaches, but many interventions do not systematically incorporate such techniques or draw on evidence and theory related to behaviour change.

Current therapeutic and behaviour change interventions can be described as 'complex interventions'; that is, they consist multiple independent and interdependent components. The UK Medical Research Council (MRC) framework [12,13] provides guidance for how 'complex' (i.e. multifaceted) interventions should be developed and evaluated, highlighting in particular the issues that should be addressed as part of the *intervention development*. Specifically, the framework recommends: *establishing evidence *(about the problem and possible solutions), *identifying or developing a testable theoretical model *(of the problem and the solutions) and *modelling the pathways through which the intervention is hypothesised to influence the outcome*. These aspects can be addressed through cycles of iterative and summative work. Few current interventions to improve children's participation in physical play and leisure have been developed in this systematic way. The models about how they may affect the outcomes are limited and, to our knowledge, there has been no empirical modelling of the possible causal pathways.

The present project will use the MRC guidance [12,13] and an integrated therapeutic-behavioural approach (described below) to develop a multifaceted theory- and evidence-based intervention to increase what children *can do *and what they *actually do *in terms of physical play and leisure.

### The theoretical framework: an integrated therapeutic-behavioural approach

**Figure 1:**
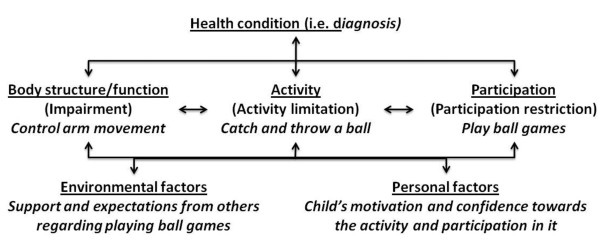
**An illustrative application of the ICF: children with motor impairments and playing ball games**.

It is widely acknowledged that the traditional biomedical model of illness does not sufficiently explain illness and health, particularly in children with motor impairments, as their health and quality of life are related to multiple factors [14]. The WHO developed the International Classification of Functioning, Disability and Health (ICF) framework [15] that incorporates biological, individual and social perspectives as components of illness and health. The core components of the ICF are impairment (of bodily structure or function), activity and activity limitations (i.e. what one '*can do*' or cannot do in a standardised environment) and participation and participation restrictions (i.e. what one '*actually does*' in real life situations). The ICF has been used widely in research and practice in relation to chronic conditions, and there is an adapted version of the ICF for Children and Youth (ICF-CY [15]). The application of ICF to children with motor impairments and a physical play/leisure (ball games) is illustrated in Figure 1.

In children with disabilities, the factors explaining variation in *participation *are likely to include: impairment (e.g. reduced strength, pain);[16,17] activity limitations (e.g. difficulties in catching and throwing);[18] personal factors (e.g. the child's preferences, emotions and motivation); [18] and environmental factors (e.g. the parents' preferences and behaviour, the parents' and school's perceptions of the child's problems, and the child's region of residence) [17-19]. 'Health condition' (e.g. cerebral palsy) is unlikely to explain significant variation in participation [20]. The relevance of the different factors in predicting participation is likely to depend on the nature of the pursuit or play [16,17]. Without rigorous research, it is unclear how the predictors relate to children's participation in physical play and leisure.

In improving participation, empirical evidence [10,11] has shown that the application of ICF components can be significantly strengthened by conceptualising activity and participation as behaviour, and subsequently integrating beliefs and behavioural regulation strategies related to the behaviour into the 'personal factors' component of the ICF. For example, a child's confidence ('a belief about capability'[21]) in catching and in playing ball games with others is likely to influence whether the child *can *catch a ball (a behaviour) and whether the child *actually plays *ball games with others (a behaviour). For children with motor impairments, intervening to change behaviour has been found to be more effective than intervening to change impairments [22].

There is thus a strong argument for using an integrated therapeutic-behavioural approach [23] to develop interventions aimed at increasing children's participation. This approach provides an interdisciplinary, evidence-based conceptual framework for identifying potential predictors of children's participation, and provides a way of making explicit the currently implicit theories behind many non-drug interventions for children with participation problems.

## Methods/Design

The overall methodology is based on the MRC guidance [12,13] for developing and evaluating complex interventions. The focus will be on the 'developing the intervention' phase, operationalised similarly to previous studies [e.g. [24]] (Table 1) as five main steps:

**Table 1:** Steps used to operationalise the MRC framework and their application in this work

**Steps in previous work**^**24**^	Steps and research questions in this work
1: Identify mediators of change	1a-c: What are the likely predictors (e.g. impairments, beliefs) of physical play and leisure that could be targeted to increase children's activity and participation?

2: Select (or develop) a theoretical model	2: How do the proposed predictors relate to each other and to activity limitations and participation restrictions in physical play/leisure?

3: Select intervention techniques and specify pathways to change	3a-b: What intervention components (i.e. 'therapeutic activities' and behaviour change techniques) could be used to target the proposed predictors, and what are the hypothesised pathways from these strategies to participation?

4: Operationalise the intervention	4: How can the intervention components best be operationalised so that the intervention is acceptable (to children, parents and professionals) and feasible?

5: Conduct a modelling experiment	5: Which of the intervention components are most likely to have an effect on the outcome(s), and what are the interactions between the components?

1) identify (a) biomedical, (b) personal and (c) environmental factors proposed to predict children's participation in physical play and leisure;

2) develop an explicit model of the key predictors of participation;

3) select (a) therapeutic and (b) behaviour change strategies to target the proposed predictors, and specify the pathways through which the strategies are hypothesised to change participation;

4) operationalise the strategies in a feasible and acceptable intervention; and

5) model the intervention processes and outcomes within single cases.

The primary outcome will be children's participation intensity (i.e. frequency divided by number of pursuits). The outcome has been specified [25] in terms of its target (the child), action (participation in physical play and leisure), context (the child's life outside school curriculum) and time (the past four months). The study has been approved by the North of Scotland Research Ethics Committee 1.

### Population

Children with motor impairments and participation restrictions referred to occupational therapy or physiotherapy will be approached. Inclusion criteria will be: (i) presence of at least one problem in body structure or function (e.g. muscle tone, body awareness) as identified by a paediatrician or a therapist; (ii) can mobilise independently (with or without aids); (iii) aged 6-8 years.

The age criterion was selected as the research team's experience suggests that children in this age range engage in pursuits and play that are sufficiently similar for them to be targeted using the same intervention; and because there is a valid and reliable outcome measure for these children's participation [26]. There will be no criteria for diagnosis or cognitive level; descriptive data about these characteristics will be collected and included in the analysis as independent variables. The inclusion criteria regarding age and cognitive level may be adjusted as further evidence emerges.

### Sampling, data collection, and data analysis

The methods for sampling, data collection and data analysis will be specific to each step as described below. Informed written consent will be taken from parents, and informed assent will be negotiated with the children on an on-going basis (please see 'Ethical considerations' below).

#### Step 1a: Identify impairments that predict children's participation in physical play and leisure

A survey involving children who meet the inclusion criteria will be undertaken. The sample will consist of children from six therapy services within NHS Scotland and England. Data about participation will be collected by therapists at first appointment in the occupational therapy or physiotherapy service using CAPE [26] (physical pursuits only), a child-friendly, interviewer-administered questionnaire.

Data about impairments and activity limitations will be collected from therapists' routine observations of the child (e.g. standardised assessments, clinical observations) at the initial assessment. Therapists will be asked to provide their observations alongside the child's participation data. The data will subsequently be coded using the ICF-CY [15]. Multiple regression will be used to identify the impairments most strongly related to limitations in activity and participation in physical play and leisure.

The target sample size is approximately 280 children. This is determined by a combination of requirements of the multiple regression analysis and feasibility. The regression analysis will include approximately 29 independent variables related to impairment (e.g. motor organisation and planning, tactile functions, mobility of joints), environment (e.g. beliefs about the child's health condition;[27] and beliefs about consequences of, capabilities in, and social influences [21] regarding participation in the target pursuit or play - see step 1c for details about data collection) and the child's personal factors (e.g. age, health conditions). Taking the larger of 104 + *m *or 50 + 8*m (where *m *is the number of independent variables)[28] indicates that a minimum sample size required will be 282 children. In terms of feasibility, it should be possible to collect data from 288 children in 4 months based on the following assumptions: involve 5 NHS occupational therapy and physiotherapy services; conservatively estimate 15 therapists per service;[29] with 60% of therapists agreeing to collect data; [30] each therapist seeing on average 2 new children a month [29] who meet the inclusion criteria; and 80% of parents [30] and children agreeing to anonymised data collection.

#### Step 1b: Identify children's beliefs about participation in physical play and leisure

'Child-friendly interviews' will be conducted with a sub-sample of children participating in step 1a. All children willing to participate will be included, with a target sample size of 25. Techniques similar to those used in other interview studies [31,32] with young children (age 4+) with health problems will be adopted. This will involve 'mapping' with visual-grids [33] and include the use of creative techniques such as representations of various participation contexts, picture prompts, free drawing and a character that the child can associate with. The techniques will be used to encourage the child to construct stories about participation in physical play and leisure. The children will be active participants in the 'story' construction, with the idea that this will allow them to project their experiences and beliefs in a non-intrusive, child-led manner. The techniques will require minimal motor skills and the children will be free to choose techniques that they wish to use. The interactions will be recorded and transcribed, and analysed using the framework approach,[34] with the integrated ICF as the coding framework.

#### Step 1c: Identify parents' beliefs about their children's participation in physical play and leisure

A survey of parents of children participating in step 1a will be conducted. This will build on the existing evidence and mapping of this evidence onto psychological theories about health-related behaviour (e.g. the Common Sense Self-regulation Model [27,35]). A questionnaire, similar to those commonly applied in behavioural studies, will be used to investigate parents' beliefs about their child's problems and their child's participation in physical play and leisure. A multiple regression analysis will be used to investigate relationships with the parents' beliefs and their child's participation.

#### Step 2: Develop an explicit conceptual model of the key predictors of participation

Evidence from 1a-c will be synthesised with existing evidence and theory to develop a further, more specific model. This will include specifying the proposed predictors (e.g. 'motor control' in impairments; 'confidence' in child's beliefs; and 'consequences of the child's condition' in parents' beliefs) and the hypothesised pathways between each of the proposed predictors and children's activity and participation.

#### Step 3a: Select 'therapeutic strategies' for targeting the proposed predictors

A focus group will be held with 7-8 senior occupational therapists and physiotherapists, identified through professional specialist interest networks. Therapists will discuss and classify intervention components extracted from current practice- and literature-based treatment manuals according to whether or not they would be used to target the specific predictors. Therapists will be asked to suggest any additional strategies not included in the existing list, and to discuss the assumptions underlying the hypothesised relationships between the various strategies and the predictors.

#### Step 3b: Identify behaviour change strategies for targeting the proposed predictors

Specific strategies will be identified from the matrix of behaviour change techniques [36] that recommends which techniques should be used for a given predictor. Each technique will be specified using the taxonomy of behaviour change techniques [37] (or a more recent taxonomy if available) and the relationships between the techniques and the predictors will be specified using the theoretical underpinnings related to each technique.

#### Step 4: Operationalise the intervention strategies in a feasible and acceptable intervention

The intervention strategies will be operationalised and their mode, context and frequency of delivery specified through consultation with therapists, experts in behaviour change (members of the Aberdeen Health Psychology Group), children and parents. Advice on the frequency and intensity with which the different strategies should be delivered will be sought from the therapists and behaviour change experts. Advice on acceptable and feasible modes, contexts and frequencies of delivery will be sought from the children, parents and therapists.

Steps 3 and 4 will culminate in an intervention manual that carefully specifies the population likely to benefit from the intervention (including age, impairments, personal and environmental dimensions), as well as the intervention content, frequency and intensity, and mode and context of delivery. This will involve further investigating different outcome measures, including self-report (e.g. CAPE, [26]. Canadian Occupational Performance Measure [38]) and objective measures (e.g. accelerometers), and developing a rigorous yet feasible protocol for outcome measurement.

#### Step 5: Model the intervention processes and outcomes within single cases

Three experimental single case studies, using a mixed methods approach, will be used to: pilot the intervention protocol; explore effects of the intervention and its components within a child; and further explore the acceptability of the intervention to children, parents and therapists. The design will allow exploration of any aspects of intervention delivery where there is more than one obvious option (e.g. to explore the most feasible mode and context of intervention delivery). Single patient interrupted time series design,[39] that is repeated measures at baseline and follow-up, will be used to estimate intervention effects and potential biases in these estimates. Semi-structured interviews with children (using similar methods to those used in step 1), their parents and therapists will be used to explore acceptability. Quantitative data will be analysed using an appropriate time series statistical method (e.g. time series regression) and qualitative data using a framework analysis [34]. At the end of step 5, revisions to the protocol will be made, resulting in a final intervention protocol ready for a formal effectiveness study (e.g. randomised controlled trial).

### Ethical considerations

The current understanding of childhood suggests that children both actively generate their own worlds [40] and are influenced by their environment [41]. This includes an understanding that different children experience the world differently [42]. There is also an appreciation that children have rights, two of which are of specific importance when considering research with children: (1) a right to make a contribution, and (2) a right to be safe from harm.

The intervention that will be designed during this programme of work is aimed to be of benefit to children with motor impairments. For this to succeed, it will be essential to gain knowledge of these children's views and perceptions about participation in physical play and leisure. Involving the children themselves helps to maximise the likelihood that the intervention will address the right issues and be relevant and acceptable to children with whom it will be used. In research involving children, special considerations need to be given to ethical (and related scientific) issues. These are in addition to the usual ethical and scientific considerations required. To acknowledge this, we have agreed upon principles that we will used to guide the conduct of this project. These are, in brief:

■ children will be respected and appreciated as important contributors without whom the project would have limited meaning;

■ active steps will be taken in designing and conducting the research to ensure that children will be empowered to participate on their own terms, including expressing views on the ways in which they wish or do not wish to contribute;

■ as part of empowering children, research methods that are 'child-friendly' and 'in-tune' with children's ways of experiencing the world will be used; and

■ good relationships and trust will be proactively built with both the children and their carers, to reduce any anxiety or worry about the children's participation.

One aspect of implementing these principles will be an involvement of a child advisory group in conducting the project. The group consists of five children with motor impairments, aged 4-9 years (ages at the start of the study). The study has research ethics governance approval (ref: 11/S0801/2 with further application to be submitted for step 5).

## Discussion

The project described here will develop an intervention to increase participation in physical play and leisure in children with motor impairments. The main output will be an intervention manual that is based on empirical evidence and on a theoretical model of disability as behaviour, and is clearly defined, feasible for use in practice and acceptable to children, families and professionals. If the intervention is found to be effective in a subsequent formal evaluation, the intervention will support children with motor difficulties in attaining life-long well-being and participation in society.

The findings will have high relevance for children with motor impairments, their families, and for therapists working with them. Evidence about the likely predictors of participation in physical play (step 1) will provide guidance to parents and therapists about the key factors to consider when assessing children's participation. The hypotheses about the mechanisms through which the predictors may relate to the outcome (step 2), will support therapists to develop evidence-based hypotheses for practice about how the interventions they use and recommend may have an effect. In combination, steps 1 and 2 will thus allow families and therapists to make more informed decisions about where to focus their resources at assessment (i.e. which factors to assess) and intervention (i.e. which factors to target).

The specification of the intervention strategies and their relationships to intervention targets (step 3) will improve clarity of current intervention techniques and their proposed pathways of change. This will allow therapists to describe explicitly their interventions, and the rationale underpinning them. The results will also provide therapists guidance about the issues related to acceptability of interventions from children's and parents' point of view (step 4).

The project will also have impact for theory and research in children's participation. The project will investigate children's participation within a therapeutic-behavioural theoretical framework, and explicitly draw on the two bodies of literature and evidence in developing the intervention. To date, these two approaches rarely have been applied together systematically within one study. Yet, activity and participation are major outcomes for both approaches and it is plausible that the two approaches are complementary. Specifically, it may be that the therapeutic approach will be particularly useful for investigating the 'body structure/function-activity-participation' pathway; the behavioural for investigating the 'personal factors-activity-participation' pathway; and the two in combination for investigating the 'environment-activity-participation' pathway. Combining these two approaches to develop one intervention may allow activity and participation to be targeted through several pathways simultaneously, and thus maximise the effectiveness and efficiency of such an intervention. The project presented here will provide evidence about the benefits and challenges related to integrating the two approaches in developing interventions aimed at activity and participation.

Finally, the methods described will be an example for future work in developing clearly specified non-drug rehabilitation interventions; and the model of participation in physical play and leisure (step 2), along with the clear specification of the different intervention techniques (steps 3), will provide theoretical and empirical bases for evaluation of the causal mechanisms underpinning interventions targeted at children's participation.

## Competing interests

The authors declare that they have no competing interests.

## Authors' contributions

NK proposed the study idea and led the design and writing of the funding application, as well as development of the study protocol. All authors made a substantial intellectual contribution to the development of the study proposal and the protocol. NK prepared the first draft of the manuscript; the other authors commented on the drafts. All authors approved the final version of the manuscript and take responsibility for its content.

## Authors' information

NK is a MRC Population Health Scientist; a specialist occupational therapist; an experienced mixed methodologist; and a member of the Aberdeen Health Psychology Group (AHPG). JF is the Head of the AHPG and a Reader in Health Services Research. CR is a Professor of Health Services Research and a Programme Director (the Health Care Assessment). CO is a Head Occupational Therapist, a research practitioner and an advisor to the College of Occupational Therapy (UK) in developmental co-ordination disorder. LMcK is a Professor of Management and a Programme Director (Delivery of Care). MK is an Associate Professor in Neurology and Neurosurgery, Rehabilitation and Sports Medicine; a Programme Leader (Paediatric Rehabilitation); and a Programme coordinator (National Research Programme on PEdiatric Rehabilitation, Netherlands). PR is a Canada Research Chair in Childhood Disability Research, Dissemination and Mentoring; a Professor of Paediatrics; and a co-founder of CanChild Centre of Childhood Disability Research.

## Pre-publication history

The pre-publication history for this paper can be accessed here:

http://www.biomedcentral.com/1471-2431/11/100/prepub
